# NTDs in the age of urbanization, climate change, and conflict: Karachi, Pakistan as a case study

**DOI:** 10.1371/journal.pntd.0008791

**Published:** 2020-11-12

**Authors:** Owais Fazal, Peter J. Hotez

**Affiliations:** 1 Rice University, Houston, Texas, United States of America; 2 Departments of Pediatrics and Molecular Virology and Microbiology, Texas Children’s Center for Vaccine Development, National School of Tropical Medicine, Baylor College of Medicine, Houston, Texas, United States of America; 3 Hagler Institute for Advanced Study at Texas A&M University, College Station, Texas, United States of America; 4 Department of Biology, Baylor University, Waco, Texas, United States of America; 5 James A Baker III Institute of Public Policy, Rice University, Houston, Texas, United States of America; 6 Scowcroft Institute of International Affairs, Bush School of Government and Public Service, Texas A&M University, College Station, Texas, United States of America; Walter and Eliza Hall Institute of Medical Research, AUSTRALIA

## Introduction: The megacity of Karachi, Pakistan

The United Nations (UN) projects that more than two-thirds of the global population will live in urban centers by the year 2050, with much of that growth expected in rising megacities, especially in South Asia [1]. There are widespread concerns that such megacities will outstrip their infrastructures related to water, sanitation, and hygiene (WASH) and become highly vulnerable to flooding and other catastrophic weather events, leading to sharp increases in urbanized and poverty-related neglected tropical diseases (NTDs) [2,3].

**Fig 1:**
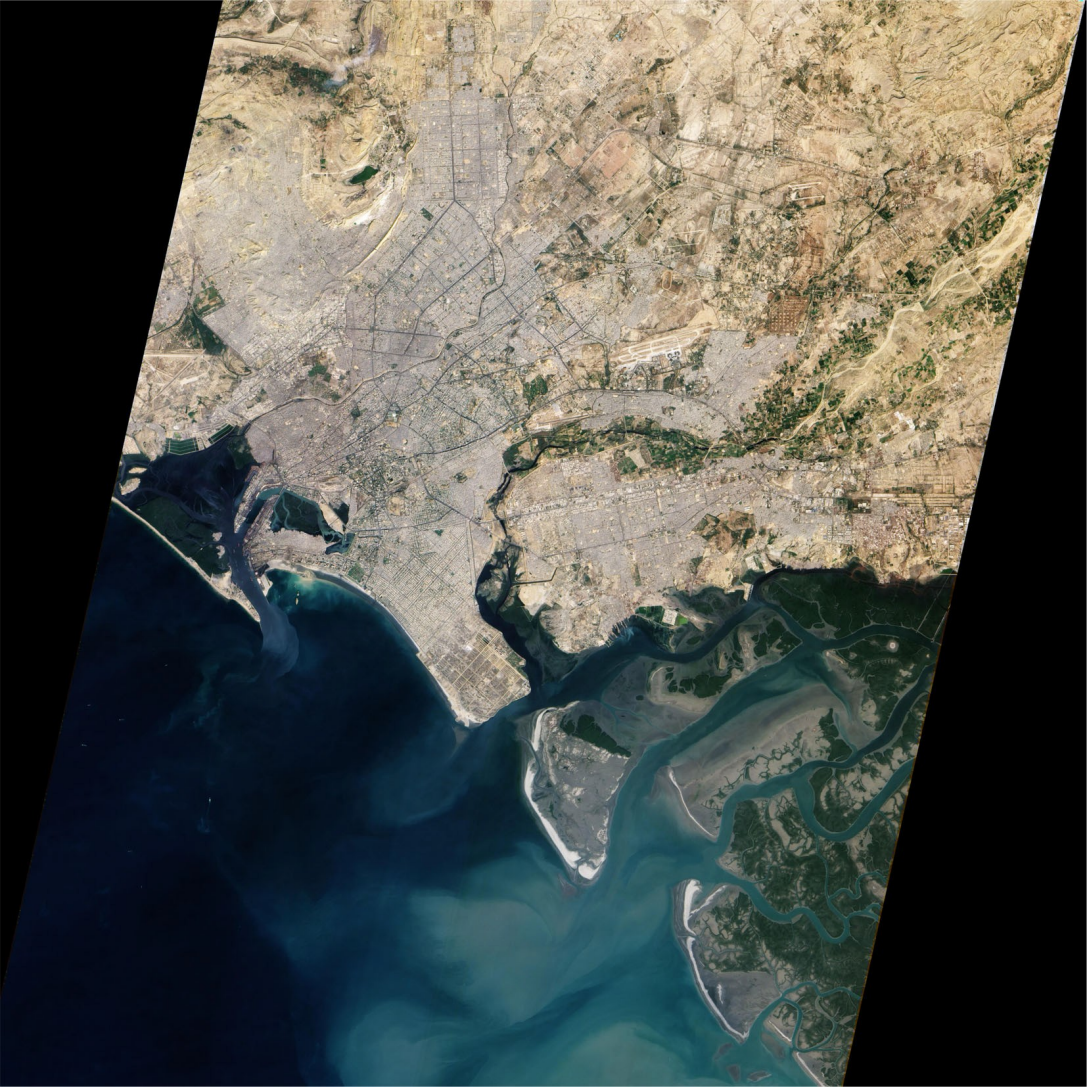
Satellite view of Karachi. *Image adapted from* http://earthobservatory.nasa.gov/IOTD/view.php?id=42538.

In this context, there are potentially valuable insights to be gleaned from the study of Karachi, Pakistan’s most populous city with a current estimated population of over 16 million people [4]. Karachi serves as Pakistan’s only major port city on the Arabian Sea (Fig 1), comprising approximately 30% of the nation’s manufacturing sector and over 20% of the national gross domestic product (GDP) [5]. It is also worth noting that the city has a large informal economy, which is not typically reflected in GDP estimates [5]. The UN projects that in the span of the next 15 years, Karachi’s population will rise to approximately 23 million people, rendering it one of the fastest growing megacities in the modern world [1]. Historically, much of the increase in Karachi’s population can be attributed to mass migrations of various ethnic groups following Pakistan’s partition from India in 1947 coupled with influxes of refugees from conflicts in nearby nations such as Afghanistan and Bangladesh [6].

A combination of factors ranging from limited healthcare infrastructure to poor sanitary conditions have likely contributed to the continued threat of diseases such as measles and polio in Pakistan [7,8]. However, for the purposes of this review, we will focus our attention on the spread of NTDs in the urban hub of Karachi.

## The confluence of urbanization, climate change, and population movement: A global perspective

It is important to contextualize the effects of urbanization, climate change, and population movement on disease in Karachi, Pakistan by briefly examining the influence of these factors on the global spread of NTDs. Several key social determinants combine with climate change to promote disease [9]. Population movements ranging from labor migration to forced resettlement following war or unrest have played a key role in the global spread of NTDs, including schistosomiasis, trachoma, and trypanosomiasis, among others [10–13]. Additionally, existing data find high burdens of NTDs in displaced populations across the globe [14–16]. In some cases, climate change–induced collapses in agriculture coupled with water and food insecurity ignite population movements to urban areas [17]. Resultant accelerated urbanization that outpaces city infrastructure development is a key factor in the endemicity of NTDs globally [9]. Simultaneously, urbanization itself can reinforce global warming [18,19]. Climate change also produces more direct effects on communicable disease due to altered temperature, rainfall, and flooding, both promoting and reducing disease transmission resulting from exposure to environmental stages of arthropod vectors, snail intermediate hosts, or larval helminths in the soil [17]. Regional climate variations in some cities have become too extreme to support certain vectors or zoonotic hosts, while major flooding in other urban centers has translocated these hosts throughout the local river system [17]. Finally, poverty remains the dominant social determinant for NTDs [9]. NTDs remain most common in settings of poverty and play a central role in perpetuating poverty through their detrimental impact on maternal and child health and human productivity [9].

## Karachi’s urbanization and the rise of informal squatter settlements

In response to the rapidly increasing demand for housing facilities across Karachi, 6 separate city development plans had been proposed between 1949 and 2006, although none of these were ultimately implemented until the Karachi Strategic Development Plan 2020 [20,21]. As a consequence of this frequently unchecked growth, Karachi continues to face a chronic shortage of housing units, electricity, clean water supply, and urban transportation facilities and services [6]. The city’s lack of affordable housing has prompted the rise in informal squatter settlements, locally referred to as “katchi abadis.” Currently, researchers estimate that around 50% of the total population of Karachi resides in these squatter settlements, which are frequently located several kilometers away from the more developed parts of the urban hub [22]. The residents of these settlements are typically poor and deprived of educational opportunities and quality public transportation systems for their daily commutes. The high rates of poverty, lack of sanitation, poor drainage systems, crowding due to unplanned spatial expansion, and limited access to healthcare facilities all contribute to the high NTD burden experienced in Karachi as well as Pakistan as a nation [6,20,23].

Karachi’s rising population density likely promotes the spread of arboviral infections, particularly in urban slums. *Aedes aegypti* mosquito vectors are well adapted to Karachi’s urban environment, resulting in higher dengue virus (DENV) and chikungunya virus (CHIKV) incidence rates in recent years [24–26]. This also leaves Karachi increasingly susceptible to future outbreaks of the Zika virus [27,28]. West Nile virus (WNV) and Japanese encephalitis virus (JEV) are also endemic arboviruses [29]. High rates of WNV detection in various livestock brought into the city such as horses highlight a major obstacle to eliminating novel WNV infections in Karachi [29,30].

Deforestation and recent irrigation projects in Karachi have given rise to breeding grounds for *Anopheles* mosquitoes and the associated spread of *Plasmodium vivax* malaria throughout the city [31]. Recent studies have also highlighted common breeding ground locations of *Culex* mosquitoes in Karachi, including newly created ground pools at the heavily populated riversides of various tributaries of the Lyari river [32]. Aggressive urbanization without proper sanitary measures has also been linked with an emergence of leptospirosis and other enteric NTDs in Karachi [33–35]. However, a positive outcome of the city’s rapid growth is the increasing number of Karachiites with access to affordable healthcare facilities, presumably leading to more frequent NTD detection and treatment [36].

## The emerging threat of climate change in Karachi

Historically, Karachi and the surrounding coastal areas of Pakistan have had a moderately arid climate with hot summers and mild winters [37]. Karachi generally receives monsoon rains from July to September, and there has not been a significant variation in terms of the timeframe of the monsoon season in Karachi over the past 25 years [37,40]. However, a key characteristic of Karachi’s climate is the often unpredictable level of rainfall on a yearly basis [40]. In addition to causing warmer temperatures in Karachi over the past few years, climate change has likely contributed to the heightened variability in monsoon rains in the city and throughout the Indian subcontinent [38,39]. According to data collected by the Pakistan Meteorological Department from 1985 to 2014, Karachi’s average annual precipitation ranges from 10 to 150 mm [41]. To illustrate this phenomenon, 2014 went down as one of the driest years on record over the past couple of decades in Karachi, with total annual rainfall falling under 50 mm [38]. This dry year stands in stark contrast to the 142 mm of precipitation Karachi received in a span of just 24 hours in July 2009 [38]. Cycles of drought and intense rainfall in the city continue to present a substantial threat to Karachiites. This uncertainty in identifying rainfall trends in Karachi also presents a vital challenge to disaster preparedness in the city.

Seasonal deviations in monsoon rainfall and associated flooding affect the severity and distribution of NTDs throughout the city. In addition to giving rise to breeding grounds for a variety of mosquito vector species, seasonal flooding often results in a substantial increase in the rodent population of Karachi [42]. These rodents can serve as vectors for the spread of leptospirosis, especially in informal squatter settlements, and serves as an example of how climate change and urbanization reinforce each other [42]. Intense rainfall after periods of drought in Karachi have also helped promote past outbreaks of cryptosporidiosis in the city [43–45]. Furthermore, drought frequently forces locals to switch to less safe supplies of water, increasing the risk for transmission of diseases such as typhoid and paratyphoid fevers [46].

Although precipitation trends remain difficult to identify in Karachi, rising mean land surface temperatures, rising sea surface temperatures, and increasing humidity levels have been documented in recent years [25,41]. A local study partially attributed the rise in dengue incidence in Karachi to all 3 of these important climatic changes [25]. Rising temperature and humidity levels in Karachi have also been linked with a marked rise in *P*. *vivax* malaria burden over the past decade [31].

It is also worth noting that the natural drainage system of Karachi comprises 3 major river systems: the Hub, Lyari, and Malir rivers. Although these river basins were once capable of diverting storm water safely to the Arabian Sea, the riverbeds have been adversely affected by urban settlements and agricultural activities [38,39]. This phenomenon has made Karachi further susceptible to the detrimental effects of monsoon season flooding.

## Ethnic strife and political conflicts: A city of migrants

Accelerating the problems of aggressive urbanization, poverty, and climate change has been political instability resulting from wealth disparities and ethnic strife in association with a rising Urdu-speaking population since the 1947 partition at the expense of Karachi’s Sindhi-speaking population [6,22,47,48]. These dramatic shifts in the demographics of the city have led to frequent political gridlock between the Mutahida Quomi Movement (MQM) and the Pakistan Peoples Party (PPP), which represent the Urdu- and Sindhi-speaking segments of the province of Sindh, respectively. The war in neighboring Afghanistan has further destabilized Karachi due to a proliferation of private militias, arms trade, and the establishment of a substantial war economy supported in part by an expanding drug trade [49].

In a survey conducted in 2009, the National Database and Registration Authority (NADRA) of the government of Pakistan reported that Karachi houses over 1.7 million undocumented immigrants, primarily consisting of Bangladeshis, political refugees from Burma, and displaced Afghans [50]. In addition to receiving such groups from neighboring countries, Karachi has received millions of labor migrants from within Pakistan, many seeking safety from Taliban presence along Pakistan’s border with Afghanistan [6,49].

There is no existing evidence that suggests that Karachi’s migrant communities have introduced novel neglected pathogens in recent years. However, local researchers have documented concerns regarding the various populations migrating into Karachi and their potential to introduce NTDs not currently endemic to the urban population [54]. A key example is lymphatic filariasis, an NTD that has remained endemic throughout several parts of India and Bangladesh [51–53]. Immigration to Karachi from these 2 nations over the past few decades has incited concerns over the introduced transmission of the disease among the indigenous population [54]. Local researchers claim that Karachi’s favorable climatic conditions coupled with current and novel sources of microfilariae make it highly susceptible to the spread of imported lymphatic filariasis [54].

In addition to addressing the potential impacts of migrant communities from outside of Karachi, careful consideration must also be given to population mobility within the city. Similar to other regions across the globe, Karachi has struggled in its *P*. *vivax* malaria elimination efforts in part due to the failures of adequately addressing local population mobility and migration [55]. Local movements between different temporary residences in the city also affect the spread of other NTDs in Karachi, including the novel emergence of dengue fever in the northwest region of the city [56]. Additionally, a study on the prevalence and risk factors of typhoid fever in Karachi found that 40% of the affected study population did not own their current place of residence [46]. This finding further highlights the increased vulnerability of displaced populations in Karachi to contracting NTDs.

**Box 1** summarizes the major NTDs documented in the megacity.

**Box 1. Summary of NTDs in Karachi**

Arboviral and other viral infections:

- Chikungunya
- Dengue
- West Nile virus
- Japanese encephalitis virus
- Crimean–Congo hemorrhagic fever
- Zika virus
- Rabies

Bacterial infections:

- Typhoid and paratyphoid fevers
- Leprosy
- Trachoma
- Leptospirosis
- Cholera

Protozoan infections:

- Leishmaniasis
- Giardiasis
- Cryptosporidium
- Entamoeba
- P. Vivax

Helminth infections:

- Echinococcosis
- Ascariasis
- Hookworm diseases
- Trichuriasis
- Toxocariasis
- Lymphatic filariasis

## Arboviral and other viral NTD infections in Karachi

After the Pakistan Federal Ministry of Health was dissolved in 2011, active surveillance measures for arboviral diseases diminished [57]. Consequently, there remain gaps in data regarding the prevalence and distribution of neglected diseases in the nation, including mosquito-borne arbovirus infections [57].

Outbreaks of chikungunya have been documented periodically in Karachi, with the most recent 2017 outbreak infecting over 30,000 individuals [24]. These outbreaks have been attributed to numerous water-filled containers serving as breeding grounds for *A*. *aegypti* in conjunction with warming temperatures from climate change [24,58]. Similar conditions have also likely influenced the spread of dengue fever in Karachi. Climatic changes including rising mean land surface temperatures, increasingly erratic rainfall patterns, and rising mean sea surface temperatures have all been linked with dengue outbreaks in the city [25]. Stagnant bodies of water near domestic premises, lack of window screens, and limited repellent use all played a substantial role in the dengue burden faced by the district of Malir in Karachi [26]. These pressing issues are compounded by the fact that many Karachiites often have limited understandings of appropriate preventive measures for dengue fever [59].

A recent cross-sectional study examining causes of arboviral diseases in Pakistan described an “active and persistent” spread of WNV through Southern Pakistan, including the city of Karachi [60]. Of the total of 997 patients enrolled in the study, 79 patients exhibited immunoglobulin M (IgM) antibodies for WNV as well as JEV, indicating a substantial prevalence of both arboviruses in the region [60]. There also proved to be a significant number of WNV–dengue coinfections, many of which were associated with neurological symptoms in affected patients [60]. Other studies have also helped confirm the presence of WNV and JEV in the mosquito vector population of the city [29,32]

A multisource outbreak of Crimean–Congo hemorrhagic fever (CCHF) was reported in Karachi in 2016, with a high case fatality rate of 75% [61]. Of the 8 confirmed CCHF cases at various health facilities in Karachi, the majority of patients were males engaged in animal trading activities in the city [61].

There have also been recent concerns regarding the potential spread of the Zika virus in Karachi due to a recent increase in Guillain–Barre syndrome cases of unknown aetiology in the Pakistani population [27,28]. Despite the fact that no clinical cases of Zika have been reported in Pakistan, it is important to acknowledge factors that may obscure Zika detection in cities such as Karachi [27,28]. Specifically, patients who present with Zika symptoms are more likely to be investigated for dengue fever by local medical practitioners, as Zika virus diagnostic tests are not currently included in Pakistan’s clinical protocols [27,28].

Karachi is also home to a large stray dog population, with thousands of dog bites occurring every year in the urban hub [62]. Multiple studies have highlighted the impact of frequent dog bites and the associated spread of rabies throughout Karachi, and 2 separate studies have evaluated general health practitioners’ and the general population’s knowledge of rabies as well as dog bite management [62,63]. Although it appears that most healthcare practitioners in Karachi are aware of rabies and its associated symptoms, these professionals are less aware of the rabies vaccine schedule as well as adequate dog bite wound care [62,64]. This lack of knowledge results in numerous dog bites going untreated every year in the city [63].

## Bacterial NTD infections in Karachi

Typhoid and paratyphoid fevers are some of the most studied NTDs present in Karachi, with some studies linking the emergence of these conditions to consumption of contaminated street food and water supplies, both of which are common practices throughout the city [46,65,66]. Low-income communities in Karachi typically lack access to piped water supplies, and these same communities often face the issue of improper drainage of sewage [46]. Water quality is variable and its delivery is disorganized, with locals often forced to pay for water vendors that supply water in reused containers [46]. Depending on the cost of the vendor, the water supplied is frequently of substandard quality and thus may increase local vulnerability to typhoid and other microorganisms [46].

Furthermore, antimicrobial drug resistance remains one of the most critical public health challenges faced by Pakistan, resulting in increasingly severe infections and complications, longer hospital stays, and increased mortality rates [67]. In Karachi, the development of antimicrobial drug resistance in *Salmonella typhi* and *Salmonella paratyphi* has presented major obstacles in terms of effectively treating typhoid patients, as local physicians often have very limited options for typhoid drug prescription [65,66]. A 2019 study conducted at a private hospital in Karachi found that over two-thirds of patients with a positive blood culture for *S*. *typhi* and *S*. *paratyphi* yielded multidrug resistant (MDR) isolates [65]. This study confirms previous findings of a high degree of MDR *S*. *typhi* and *S*. *paratyphi* in Karachi, particularly among children in urban squatter settlements [46,65,66,68].

Cases of leprosy have been documented in different locations in the city, with a past study exploring social stigmas faced by those afflicted with the NTD [69,70]. According to the study, 7.3% of the survey population knew someone who had leprosy [70]. Attitudes toward leprosy are generally compassionate, although misconceptions regarding the disease’s transmission have led to social stigma in past years, particularly when it comes to sharing food and forms of transportation such as buses [70].

An additional bacterial NTD that may have implications for future health interventions in Karachi is trachoma. While cases of trachoma have been documented in Pakistan, minimal research has been conducted on its prevalence and spread throughout Karachi [71,72]. A past study claimed that there was insufficient evidence for the spread of active trachoma in the urban population of Karachi [73]

Previous serological studies have confirmed the endemicity of leptospirosis in Karachi after testing on humans as well as cows, buffaloes, sheep, goats, and rodents [33,35]. Rising rodent populations in the wake of flooding have been linked to increased incidence of leptospirosis in Pakistan [35,42]. Flood drainage contaminated with rodent urine frequently collects on roads, resulting in a potential source of leptospirosis infection for those walking barefoot, especially children [42].

Another bacterial disease that has demonstrated seasonal periodicity during the monsoon season in Karachi is cholera [74]. Like many other enteric pathogens in endemic areas, cholera primarily affects young children in Karachi [74]. Despite cholera’s association with poverty, a study from Aga Khan University found that a sizable proportion of cholera cases in Karachi were detected in expensive private hospitals [75]. This finding suggests that problems with Karachi’s sanitation infrastructure affect people of all income levels, creating further concerns about the possible contamination of Karachi’s water delivery systems [75].

## Protozoan NTD infections in Karachi

Cutaneous leishmaniasis remains endemic throughout Karachi, and patients who display symptoms of cutaneous leishmaniasis usually visit major tertiary care referral centers in the city in order to receive treatment [76–78]. While these facilities typically document the annual number of cases received, exact estimates of incidence in surrounding areas, particularly slums and peri-urban localities, are underestimated and not well known [76,77]. Local researchers have previously suggested training female health workers as a potential ally in identifying cases of cutaneous leishmaniasis among girls and women and referring affected patients to the appropriate health facilities [76].

Giardiasis is another protozoan NTD with documented cases in Karachi [34]. A 2008 study conducted in Ghosia Colony, an urban slum of Karachi, documented that 28.9% of a sample of children aged 1 to 5 had *Giardia lamblia* present in their stool [34]. In fact, *G*. *lamblia* was the most common intestinal parasite documented in the study [34].

A number of studies conducted at hospitals in Karachi have confirmed a high prevalence of cryptosporidium among their patient populations [43–45]. One study conducted at the Jinnah Postgraduate Medical Centre found that 40% of immunosuppressed patients tested positive for cryptosporidium, with a particularly high percentage of cancer patients afflicted with the diarrheal illness [44]. Researchers have also acknowledged difficulties in detecting cases of cryptosporidiosis in Karachi on account of the symptomatic similarities with other gastrointestinal infections [43,45]. *Entamoeba histolytica* is also an enteric parasite with substantial incidence in Karachi, with peak incidence occurring during Karachi’s summer months [43].

*P*. *vivax* remains a major cause of morbidity in tertiary care settings in Karachi and throughout Pakistan [79–83]. Recent studies have also identified *P*. *vivax* as an increasingly frequent cause of severe malaria cases in Karachi, although the mortality rate for the disease remains low [80,81]. *P*. *vivax* displays a monsoon seasonal peak from July to September, and transmission continues throughout the year [79]. Antimalarial drug resistance is also an important issue in Karachi, particularly with the emerging threat of chloroquine-resistant malaria [83]. An Aga Khan University study highlighted that physicians in Karachi continue to inappropriately prescribe chloroquine despite documented resistance [83].

## Helminth NTD infections in Karachi

Urbanized helminth infections affect a substantial proportion of individuals living in Karachi, particularly children in low-income settings [34,84,85]. Researchers previously reported a 52.8% prevalence of intestinal parasitic infections among young children in the Ghosia Colony slum [34]. The major soil-transmitted helminth infections include ascariasis, trichuriasis, and hookworm infections [34]. A high prevalence of ascariasis and hookworm infections has also been documented in schoolchildren through Karachi [85]. Interestingly, schoolboys were found to be more likely to become infected with the intestinal parasites as compared to schoolgirls, presumably due to increased outdoor exposure [85]. Living in rented housing was also identified as one of the key risk factors for contracting intestinal parasitic infections [34].

Hydatid disease is also endemic, and there are several risk factors specifically in Karachi that may increase exposure to the disease [86–88]. Livestock rearing is a common practice in several parts of Karachi and surrounding rural areas, with many households owning multiple livestock [86,87]. Moreover, possibly contaminated fruits and vegetables are regularly sold on Karachi’s streets, and limited knowledge regarding echinococcosis and its modes of transmission may further contribute to its spread in the urban hub [87]. A serological study on the dog population in Karachi confirmed a 45.4% infection rate of *Toxocara canis*, highlighting the potential threat and endemicity of toxocariasis [89]. Concerns have also been expressed about the transmission of lymphatic filariasis in Karachi [54].

## Concluding statement

The confluence of accelerated urbanization, climate change, political instability, and poverty in Karachi could create a toxic mix to promote the rise in NTDs and other infections. Our concern is that this situation is not unique to Karachi, but instead may represent a new 21st theme common to megacities across the Global South—Asia, Africa, and Latin America. While great strides are being made to control or even eliminate many NTDs through programs of preventive treatments, mass drug administration, and in some cases, vector control and case detection and treatment, the forces now in play in Karachi and other global megacities could undermine those successes. Further adding to these concerns is the emergence of Coronavirus Disease 2019 (COVID-19) in Karachi and its potential impact on reducing current disease prevention activities, including routine childhood immunization and NTD control [90]. Therefore, as we advance into this new century, we may need to consider new strategies and a revised roadmap for combating NTDs in an increasingly interconnected global environment.
